# Gray whale density during seismic surveys near their Sakhalin feeding ground

**DOI:** 10.1007/s10661-022-10025-8

**Published:** 2022-10-18

**Authors:** Glenn Gailey, Mikhail Zykov, Olga Sychenko, Alexander Rutenko, Arny L. Blanchard, Lisanne Aerts, Rodger H. Melton

**Affiliations:** 1 Cetacean EcoSystem Research, Olympia, WA 98512 USA; 2 JASCO Applied Sciences Ltd, Dartmouth, NS B3B 1Z1 Canada; 3 Far East Branch of Russian Academy of Sciences, V.I. Il’ichev Pacific Oceanological Institute, Vladivostok, 690041 Russia; 4 Blanchard Ecological, North Pole, AK 99705 USA; 5 LAMA Ecological, Anchorage, AK 99502 USA; 6 ExxonMobil Exploration Company, Spring, TX 77389 USA

**Keywords:** Western gray whale, Seismic survey, Distribution, Sound exposure levels, Vessel activity, Disturbance

## Abstract

Oil and gas development off northeastern Sakhalin Island, Russia, has exposed the western gray whale population on their summer-fall foraging grounds to a range of anthropogenic activities, such as pile driving, dredging, pipeline installation, and seismic surveys. In 2015, the number of seismic surveys within a feeding season surpassed the level of the number and duration of previous seismic survey activities known to have occurred close to the gray whales’ feeding ground, with the potential to cause disturbance to their feeding activity. To examine the extent that gray whales were potentially avoiding areas when exposed to seismic and vessel sounds, shore-based teams monitored the abundance and distribution of gray whales from 13 stations that encompassed the known nearshore feeding area. Gray whale density was examined in relation to natural (spatial, temporal, and prey energy) and anthropogenic (cumulative sound exposure from vessel and seismic sounds) explanatory variables using Generalized Additive Models (GAM). Distance from shore, water depth, date, and northing explained a significant amount of variation in gray whale densities. Prey energy from crustaceans, specifically amphipods, isopods, and cumaceans also significantly influenced gray whale densities in the nearshore feeding area. Increasing cumulative exposure to vessel and seismic sounds resulted in both a short- and longer-term decline in gray whale density in an area. This study provides further insights about western gray whale responses to anthropogenic activity in proximity to and within the nearshore feeding area. As the frequency of seismic surveys and other non-oil and gas anthropogenic activity are expected to increase off Sakhalin Island, it is critical to continue to monitor and assess potential impacts on this endangered population of gray whales.

## Introduction

Oil and gas were discovered off the northeastern coast of Sakhalin Island, Russia, in the late 1970s. However, oil and gas development in the area did not occur until the late 1990s. Since then, a number of seismic surveys have been conducted, four offshore oil platforms and associated pipelines have been installed, and drilling activities have taken place from the platforms since they were commissioned. As part of oil and gas operations, seismic surveys are required for exploration of resources as well as to monitor oil and gas reservoirs to efficiently extract the resources. Seismic surveys use air guns to map deposits beneath the seafloor. Marine seismic surveys and vessel traffic are major contributors to global ocean noise (Hildebrand, 2009; Nowacek et al., 2015; Silber et al., 2021) and have the potential to impact marine life (Carroll et al., 2017; Richardson et al., 1995; Southall et al., 2007; Weilgart, 2013).

Overlapping with or in close proximity to the oil and gas operations off northeastern Sakhalin is the feeding ground, consisting of a nearshore and an offshore feeding area, of an endangered population of western gray whales (*Eschrichtius robustus*) that forage there each summer and fall (Cooke et al., 2018). As capital breeders, gray whales depend on the energy gained during a foraging season to sustain them during migration to and from their winter breeding grounds as well as time spent on the breeding grounds. Little information was known about the western population in the late 1990s, when oil and gas exploration and development activities commenced in proximity to their feeding areas. Consequently, several research programs were initiated to better understand and monitor the gray whale population, which included research on their movement, behavior, distribution, prey, population, and genetics (Demchenko & Fadeev, 2011; Gailey et al., 2007, 2016; LeDuc et al., 2002; Lowry et al., 2018; Muir et al., 2015a, 2015b, 2015c, 2016; Weller et al., 2002). The increased knowledge about this endangered population of whales has improved mitigation and monitoring strategies over time to minimize potential impacts of oil and gas activities on the population (Aerts et al., 2022; Bröker et al., 2015; Johnson et al., 2007; Nowacek et al., 2013).

In 2015, two oil and gas operators in the region conducted seismic surveys with up to four source vessels near the western gray whale foraging ground. Prior to 2015, the impacts of three seismic surveys (1997, 2001, and 2010) near this population have been assessed with varying results. From studies undertaken in 1997, gray whales altered their swimming speeds, orientations, respiration patterns, and distribution in response to seismic surveys (Würsig et al., 1999). Likewise, gray whale behavior and distribution responses were observed during a seismic survey in 2001 (Gailey et al., 2007; Yazvenko et al., 2007). However, a 2010 seismic survey found fewer distribution and behavioral responses and it remained unclear if the mitigation measures were effective in reducing potential impacts, if more individuals habituated/tolerated the activities, or if the studies in 2010 suffered from limited sample size, as suggested by the authors (Gailey et al., 2016; Muir et al., 2015a, 2016). Recognizing the need for additional research, one operator conducted an extensive research program in 2015 to more closely examine changes in distribution and behavior of whales exposed to sounds generated from seismic surveys and vessel activities (Aerts et al., 2022). Aerts et al. (2022) summarize the mitigation and monitoring strategies. Gailey et al. (2022) examine gray whale behavioral responses to seismic and vessel activity. The results of the study found multiple indicators of movement and respirations responses, at least in the short-term, relative to both vessel and seismic sounds (Gailey et al., 2022). In this study, we explore potential changes in western gray whale habitat utilization of the nearshore feeding area by exploring factors that may affect gray whale density estimates within the observation areas. We further explore gray whale habitat use relative to several benthic prey species in the nearshore feeding area (Blanchard et al., 2022a, b). The results of these studies provide valuable input to larger bio-energetic frameworks that predict how different levels of acoustic disturbance could affect foraging efficiency and ultimately vital rates, such as reproductive success and survival (McHuron et al., 2021; Pirotta et al., 2018, 2021; Schwarz et al., 2022b).

## Methods

### Study site, observation stations, and seismic surveys

**Fig. 1:**
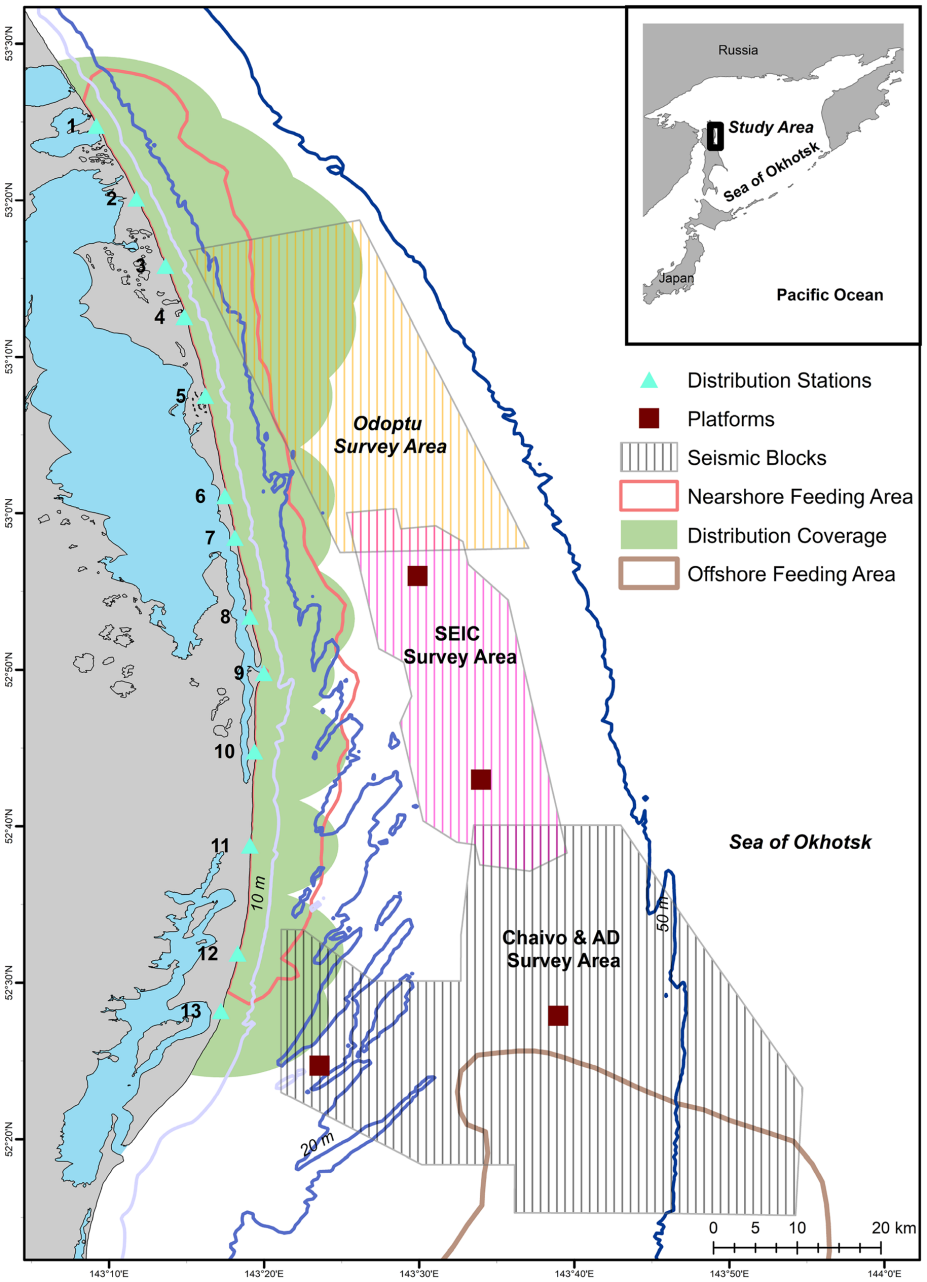
Map of the study area illustrating the 95% kernel contour of western gray whales (red line) based on 2002-2013 nearshore and offshore distribution data (see Muir et al., 2015b), the 13 shore-based monitoring stations, and the 2015 observer spatial coverage (based on station height, green polygon). Oil platforms (brown squares) and seismic survey areas (lined polygons) where activities were conducted in 2015 are also illustrated

The foraging ground of western gray whales is in coastal waters of the Sea of Okhotsk off northeastern Sakhalin Island, Russia. The nearshore feeding area, which is the focus of this study, is mostly sand substrate with a gradually sloping continental shelf with water depths usually less than 20 m within 5 km from shore (Fig. 1). Gray whale distribution and abundance were monitored from 13 shore-based stations from 1 June to 30 September 2015. The shore-based stations were spatially separated by ~10 km, covering ~122 km of coastal habitat that encompassed the known western gray whale nearshore feeding area based on aerial, vessel, and shore-based distribution data (Muir et al., 2015b). The shore-based observation height ranged from 5.6 to 32.2 m, which resulted in varying observation ranges to detect gray whales (approximately 3.8–12.4 km out to the 0.1 reticle, Fig. 1). Due to the relatively low elevations at some of the onshore stations, wooden towers (4 m height) were custom built at seven stations (4, 5, 8, 9, 10, 12, and 13) to increase the observation range. The resulting observation elevations allowed detection of gray whales > 5 km from shore, which was considered sufficient to cover the known nearshore feeding area. Despite the ability to detect whales farther from shore, gray whales have previously been observed at a mean of ~1.5 km from shore in water depths less than 20 m (Gailey et al., 2007; Muir et al., 2015a, b, 2016; Yazvenko et al., 2007). Mother-calf pairs, lone calves, and younger individuals (< 5 years of age) tend to be closer to shore, usually less than 500 m from shore (Sychenko, 2011).

During the summer of 2015, a total of four seismic source vessels operated off northeastern Sakhalin to capture data about oil and gas reservoirs within four license blocks near or within the gray whale feeding areas (Fig. 1). Seismic operations started on 6 June and ended on 23 September 2015. Seismic operations varied spatially and temporally. The two operating companies coordinated their activities with the intent to acquire seismic lines closest to the feeding areas as early in the feeding season as possible. Aerts et al. (2022) summarize the seismic operations as well as the mitigation and monitoring approaches taken in 2015 by one of the operators.

### Distribution and abundance data collection and processing

A total of 13 distribution teams, with a minimum of two people per team (total of 26 observers), conducted hourly scans for gray whales, weather permitting, at each of the 13 shore-based stations. Scans were separated by a minimum of an hour based on autocorrelation analyses indicating the number of whales in a scanned area being independent after an hour past from the previous scan. Scan sampling techniques were employed under varying weather conditions but with good visibility and Beaufort Sea State < 5. The two observers, equipped with 7×50 Fujinon FMTRC-SX reticle binoculars, at each station conducted a scan sample by continuously scanning from the northern to the southern part of their observation area at a constant rate of approximately 9.33 °/min. Due to variation in the coastline, some stations covered a slightly larger spatial area, resulting in a longer scan duration when maintaining the constant scan rate. The general scan duration (range: 18.3–21.1) was around 19.3 min (i.e., 180°/9.33°/min = 19.29 min). The software system Pythagoras (Gailey & Ortega-Ortiz, 2002) was used to conduct scans, which verbally informed observers via text-to-speech at what magnetic bearing they should be at every 10° during the scan to be as systematic as possible.

During each scan, all sightings of vessels and marine mammals, with the exception of largha seals (*Phoca largha*) and ringed seals (*Phoca hispida*), were recorded. For each sighting, the number of animals or vessels, angular distance between the animal(s) or vessel and the horizon (based on binocular reticles), magnetic bearing, estimated heading, and observer estimated distance from the station were recorded. Environmental conditions were recorded immediately prior to conducting a scan; these included date, time, station, Beaufort sea state, visibility, observer estimated swell height, percentage of glare, glare angles, and cloud cover. A weather device (Kestrel 4500) recorded air temperature, atmospheric pressure, wind speed, and wind direction prior to each scan and every 5 min for the entire observation day. Tide height data, collected as part of the seismic survey operations, were obtained post-hoc and included as an environmental variable used to adjust observation height in the distance approximation of sightings. Visibility condition was classified based on four categories: 1) Excellent conditions with clear horizon line, 2) Good conditions with little to no haze and/or rain with relatively clear horizon line, 3) Fair with some haze and/or rain but horizon still visible enough for reticle estimation, and 4) Poor with no visible horizon due to fog and/or rain. Scan sampling was not conducted under visibility code 4 due to a lack of a horizon or reference line to reliably estimate distance. Although scans were collected in Beaufort sea states higher than 3, only scans with Beaufort scale of 3 or less were used in analyses. All distribution data were recorded into the Pythagoras database system (Gailey & Ortega-Ortiz, 2002).

Due to a non-uniform gray whale density gradient from shore, conventional estimations of detection probability from the observation stations could not be applied. Instead, a double platform experiment was conducted in 2006 that indicated a gray whale flat detection curve out to the 8 km measured. In this study, observation ranges out to the 0.1 reticle ranged from 3.8 to 12.4 km. It was assumed that the flat detection curve extended out to 12 km, which could suggest some unknown underestimation of density at farther ranges from the observation. However, historical aerial and vessel based conducted within the same day as shore-based scans have never indicated higher whale densities in these farther from shore areas. In fact, the majority of over 95% kernel distribution of whale sightings from multiple platforms are within the 20 m contour (~ 5–7 km from shore) (Muir et al., 2015a, 2015b, 2015c; Muir et al., 2015a, 2015b, 2015c; Muir et al., 2016).

Distance approximation of each sighting was estimated using reticle binocular techniques. Lerczak and Hobbs' (1998) distance approximation equation was combined with Leaper and Gordon's (2001) refraction correction to calculate the estimated distance of the sighting from the observer. Atmospheric pressure and air temperature, automatically collected by the Kestrel weather device at the time of the observation, were used for the refraction correction. The magnetic declination was estimated based on the date of the sighting and geographic location of the station using the oce r-package (Kelley & Richards, 2014). Based on the location of the station, the estimated distance to the sighting, and geographic bearing, the geographic position of the sighting was calculated using the destination equation in the geosphere r-package (Hijmans, 2017). The sighting’s perpendicular distance from shore was estimated using the gDistance function in the rgeos r-package (Bivand & Rundel, 2017).

All 13 distribution teams initiated their scan on the hour, thereby covering the entire nearshore feeding area. Observers at adjacent stations likely duplicated sightings in the overlapping scan areas, which could artificially inflate the number of animals observed in a particular area. To remove the potential double counting of whales, the overlapping areas between adjacent stations were identified and sightings from the station closest to the overlapping area were used. In other words, sightings from only one station were counted to cover the overlapping spatial area during a scan. The largest observation overlaps occurred at northern stations 2, 3, and 4, which had the highest elevations and thus largest observation ranges (Fig. 1).

### Gray whale density

Gray whale density estimation in the nearshore feeding area followed previous methodological approaches (Muir et al., 2015a, b) and is briefly summarized here. A grid (1×1 km or 1 km^2^ cells) covering the observation ranges of each of the 13 shore-based distribution stations was developed. Gray whale sightings were overlaid on the grid and summarized per cell. Sightings with reticle values lower than 0.1 were excluded from the density estimation, which essentially removed sightings of gray whales observed close to the horizon where detection probability was unknown. For each 1 km^2^ grid cell, gray whale density (${\widehat{D}}_{i,j}$) was estimated in the j^th^ grid cell that was sampled during a scan survey i as follows:

$${\widehat{D}}_{i,j}=\frac{1}{{\widehat{a}}_{i,j}{A}_{i,j}}\sum_{k=1}^{{S}_{i,j}}\frac{{n}_{i,j,k}}{\widehat{p}}$$

where ${\widehat{a}}_{i,j}$ is the estimated availability correction in grid cell j during survey i, A_i,j_ is the scanned area coverage of the cell, S_i,j_ is the number of gray whale sightings per survey i in cell j, n_i,j,k_ is the number of gray whales observed in the k^th^ sighting per survey i in cell j, $\widehat{p}$ is the detection probability which was set to 1 for this study based on a flat detection curve out to observation distance equivalent to the 0.1 reticle (Muir et al., 2016). The availability correction ($\widehat{a}$) estimates the probability of an animal being at the surface during the time of observation. The whale’s availability was based on the portion of time a gray whale was at the surface while an observer was scanning a particular patch of water. Surface-dive cycle data collected by Gailey et al. (2022) in 2015 were used to estimate the availability correction.

### Prey availability

The density of gray whales on their foraging grounds is likely influenced by prey energy content in the area. To determine the spatial and temporal prey availability in the nearshore area, prey biomass data were collected in 2015 where whales had been most commonly observed in previous years. Energy content was estimated by using the caloric conversion factor. Methodological details and results of benthic sampling are presented in Blanchard et al. (2022a). Benthic sampling was replicated in the nearshore feeding area over three separate periods: Period 1–19 June–15 July, Period 2–16 July–31 August, and Period 3–1 September–24 October. Interpolation of benthic energy values from spatial locations was required to estimate the energy of each benthic species group throughout the study area. We applied Inverse Distance Weighted (IDW) interpolation to seven species variables: 1) Amphipoda, 2) Isopoda, 3) Cumacea, 4) Polychaeta, 5) Bivalvia, 6) Actinoptygerii, and 7) Total energy (summation of groups 1-6). IDW was considered the better choice than spatial models of prey energy due to limitations of other spatial models (Blanchard et al., 2022b). The mean interpolated benthic energy value was calculated per grid cell for each species group and benthic sampling periods. The mean gray whale density was calculated for the same grid cells for the three separate benthic sampling periods. A Generalized Additive Model (GAM) was used to examine the relationship between benthic energy content among the different prey variables and gray whale density. The model was applied similarly as the density anthropogenic response model (see below). Gray whale density and energy values of Bivalvia, Cumacea, Isopoda, and Polychaeta were log-transformed while square root transformation was applied to total and amphipod energy values. Sampling period, grid cell identifier, water depth, and northing (UTM Y) values were included to represent temporal and spatial variables in the model. Distance from shore was excluded as a potential variable due to high correlation (Pearson’s correlation > 0.9) with water depth.

### Acoustic monitoring and sound level estimation

Vessel and seismic sounds were recorded with Automatic Underwater Acoustic Recorders (AUARs) with a frequency range of 2 to 15,000 Hz. Acoustic buoys were strategically placed at 34 locations in water depths of 10–20 m in the nearshore area. Sound propagation models, with recorded data-based correction, were used to estimate the received sound levels (16 to 1000 Hz frequency range) within each grid cell every 5 min throughout the period of distribution observations (1 June–30 September). Over 60,000 multi-band raster grid files (one per 5 min for the duration of the study) were generated that contained acoustic metrics for both continuous (vessel) and pulse (seismic) sounds. Rutenko et al. (2022) describe details of the recorded data processing and modeling of the sound level metrics used in this study.

For each grid cell, a number of acoustic metrics were calculated for different temporal periods prior to conducting a scan survey. The periods of 2 h, 8 h, 1 d, 3 d, and 7 d were chosen to examine if gray whale densities changed relative to the amount of acoustic exposure during the specified time window. Detection of change in gray whale distribution due to sound exposure is generally not immediate. As such, these time periods represent both short-term (2–8 h) and longer-term (1–7 d) responses to sound exposure. For seismic sounds, the median and maximum root mean square Sound Pressure Level (SPL), peak SPL, and cumulative Sound Exposure Level (cSEL) were calculated for each period. For vessel sounds, median and maximum SPL and cSEL were estimated for each period. A combined total of 50 (20 vessel and 30 seismic) acoustic metrics were generated for each scan that represented the different sound variables for each period for seismic and vessel sounds. Although a number of acoustic metrics were considered for inclusion in the model, many metrics were highly correlated (Pearson’s > 0.85) within each sampling period. To minimize effects of collinearity in the models, we chose the median cSEL variable as the most representative acoustic exposure metric for both vessel and seismic sounds. Seismic and vessel sounds were, however, not as highly correlated (Pearson’s correlation < 0.6) among each other within each period or between different periods.

### Gray whale density response models

In addition to the prey availability model, a separate model was developed to examine gray whale density relative to acoustic exposure from vessel or seismic activities, taking into account spatial, temporal, and environmental influences. A separate model was required since the benthic sampling period was on a much larger temporal scale (months) than the acoustic and gray whale density data. To examine spatial relationships, northing, distance from shore, and water depth were considered. Distance from shore and water depth were incorporated in separate models due to their high correlation (> 0.9). Date and time of the scan survey were considered as temporal variables that could explain variability in gray whale densities. Vessel and seismic median cSEL metrics for each time window prior (2 h, 8 h, 1 d, 3 d, and 7 d) to each scan survey in the nearshore feeding area were also considered. Beaufort sea state and visibility conditions were considered in the model as potential environmental influences. Collinearity among the covariates was examined using pair-wise Pearson correlation coefficients for all continuous variables and box-plots were used to assess non-continuous against continuous variables. A correlation coefficient larger than 0.70 warranted concern that one covariate could mask the effects of another.

GAM models were developed to examine the associations of the covariates with gray whale density. GAM models are commonly used to examine variables that may influence spatial distributions due to their flexibility to capture non-linear density-habitat relationships. GAM models also have flexible distributions that can be adopted to model overdispersion. GAM models were implemented with the mgcv R package (Wood & Wood, 2015). We used thin plated regression splines and restricted maximum likelihood (REML) to estimate parameters. Akaike’s Information Criterion (AIC) was used to determine the best set of covariates that fit the model. Standardized residual plots were inspected to assess model fit and concurvity post-hoc tests were used to evaluate potential collinearity in the final model. A concurvity value greater than 0.75 in the worst-case estimate required closer inspection of the model.

## Results

### Effort

Distribution and abundance scans were conducted from 1 June to 30 September. The total number of days that scans were conducted during this period was 1277 and varied per observation station with a mean of 98 days (range: 83–114). With distribution teams at each station, the cumulative number of scans conducted was 10042 with a combined 16817 sightings of a population of gray whales estimated to be ~175 individuals (Cooke et al., 2018). However, a number of the sightings were likely to be duplicates among overlapping observation areas. The mean number of scans collected for each observation station per day was 7.9 (range: 7.2–9.2). A total of 290 killer whales (*Orcinus orca*), a potential predator of gray whales, were sighted during this four-month study. Sighting information of other marine mammal species are not reported here. Due to the large number of shore-based teams and duration of the study, the total number of scans collected in 2015 was more than twice the number of scans collected over the previous decade (4249 scans from 2004–2013).

### Gray whale density

**Fig. 2:**
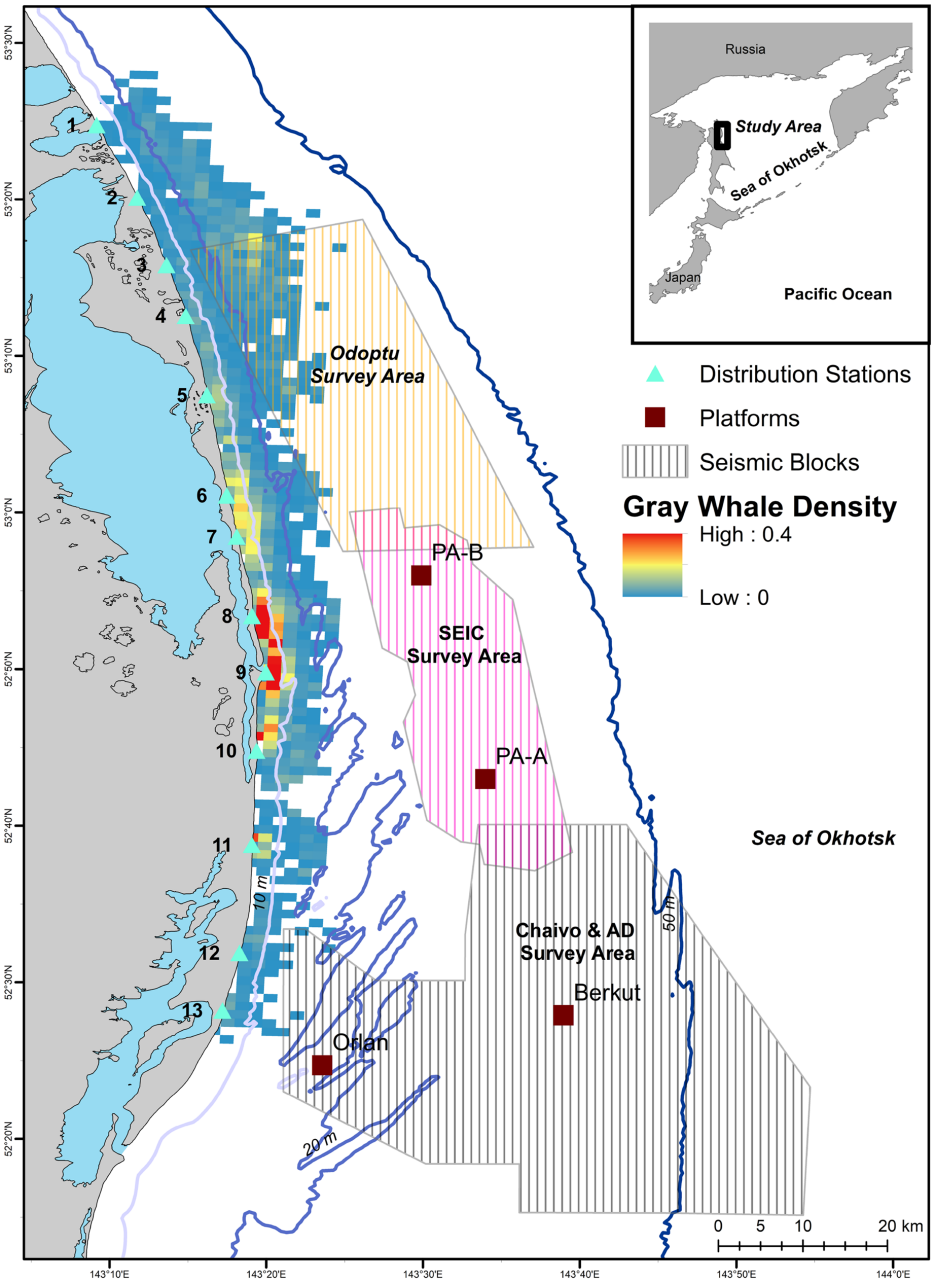
Mean density (whales/km^2^) estimates of gray whales utilizing their nearshore feeding ground off Sakhalin Island over the four-month period from 1 June to 30 September 2015

**Fig. 3:**
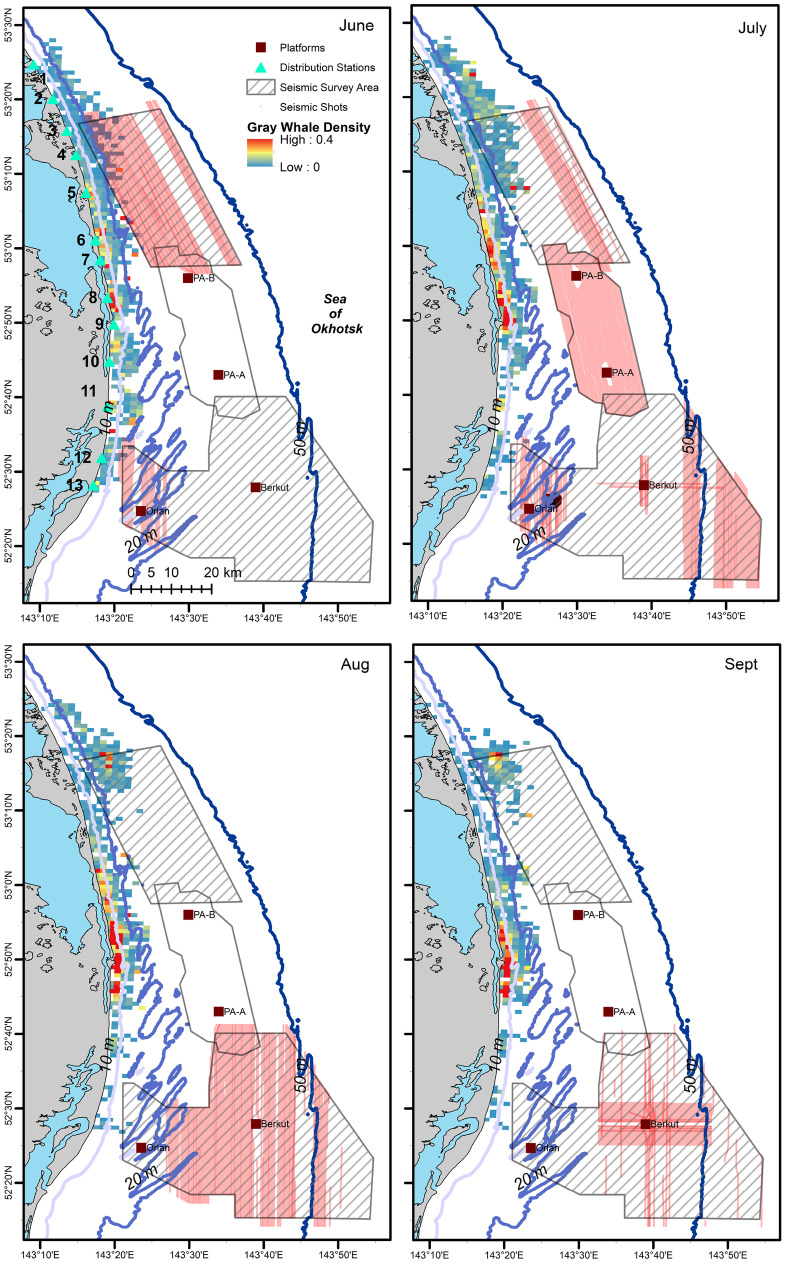
Mean monthly gray whale density (whales/km^2^) in the nearshore feeding area combined with acquired seismic lines (red lines) during the same month in 2015

Gray whales observed off Sakhalin demonstrated both consistent and variable utilization of their nearshore feeding area in 2015. For the entire four-month study, the mean gray whale density was highest around Piltun Bay mouth (located near Station 9) and ~20 km north of it (Fig. 2). Gray whale densities were also high farther from shore (~7 km, 20–30 m water depth) in the northern part of the nearshore feeding area. During the earlier part of the feeding season (June), when fewer gray whales were present, gray whales were more uniformly distributed, with the primary concentration of whales close to shore near and north of Piltun Bay mouth. Even after more gray whales arrived, the concentration of gray whales near the mouth of Piltun Bay remained throughout the field season, with whales extending their habitat utilization nearshore to areas farther north (Stations 5 and 6), particularly in July and August. Starting in July, some gray whales were observed farther from shore (> 20 m depth) in the northern part of the study area where higher densities were observed in August and September (Fig. 3).

### Spatial, temporal, and environmental influences on gray whale density

**Fig. 4:**
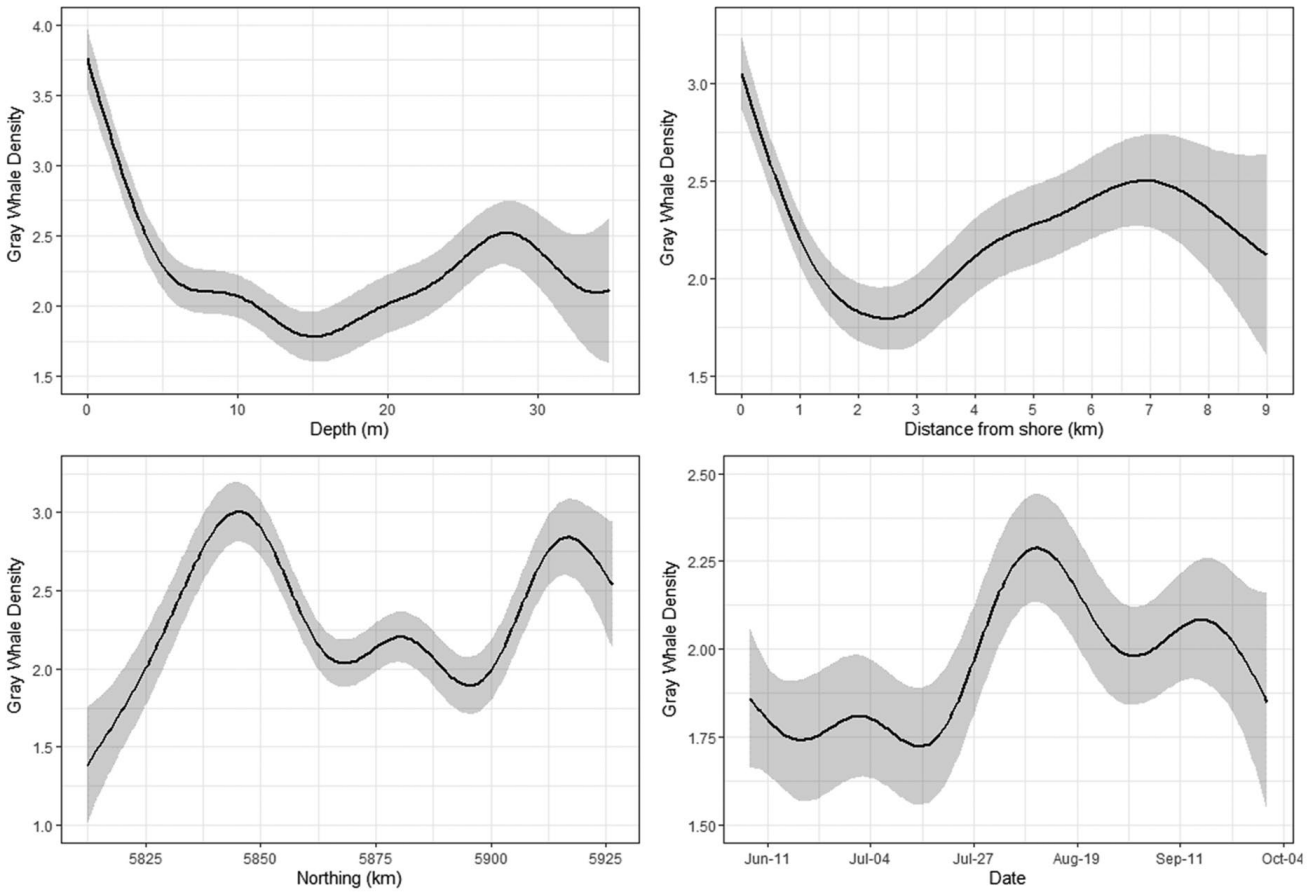
Gray whale density (whales/km^2^) response model results illustrating spatial and temporal patterns in habitat utilization of the nearshore feeding area for depth, distance from shore, northing, and date. Depth and distance from shore were highly correlated and were included in separate models

Gray whale density response models reflected distribution patterns observed in spatial plots (Figs. 2–3) with water depth/distance from shore, date, and northing explaining the most amount of variation in whale density. Since depth and distance from shore were highly correlated, they illustrate similar patterns with gray whale density decreasing rapidly to about 1.5–2.0 km from shore or ~10 m water depth. Model results also showed an increase in gray whale density farther from shore in the northern part of the feeding area as illustrated in Fig. 3. Northing patterns were similar as well, with lower gray whale density in the southern part of the feeding area, higher density in the central part (specifically around the mouth of Piltun Bay), and high density patterns observed in the northern part of the feeding area. Date was also significantly associated with gray whale density. Gray whale density increased from the start of the study (early June) until about halfway through the study period (early August), after which the density in the nearshore feeding area significantly decreased. Time of day and environmental covariates were not found to explain a significant portion of the variation in whale density (Fig. 4). Model results are summarized in Table 1.

### Gray whale density and their potential prey

**Fig. 5:**
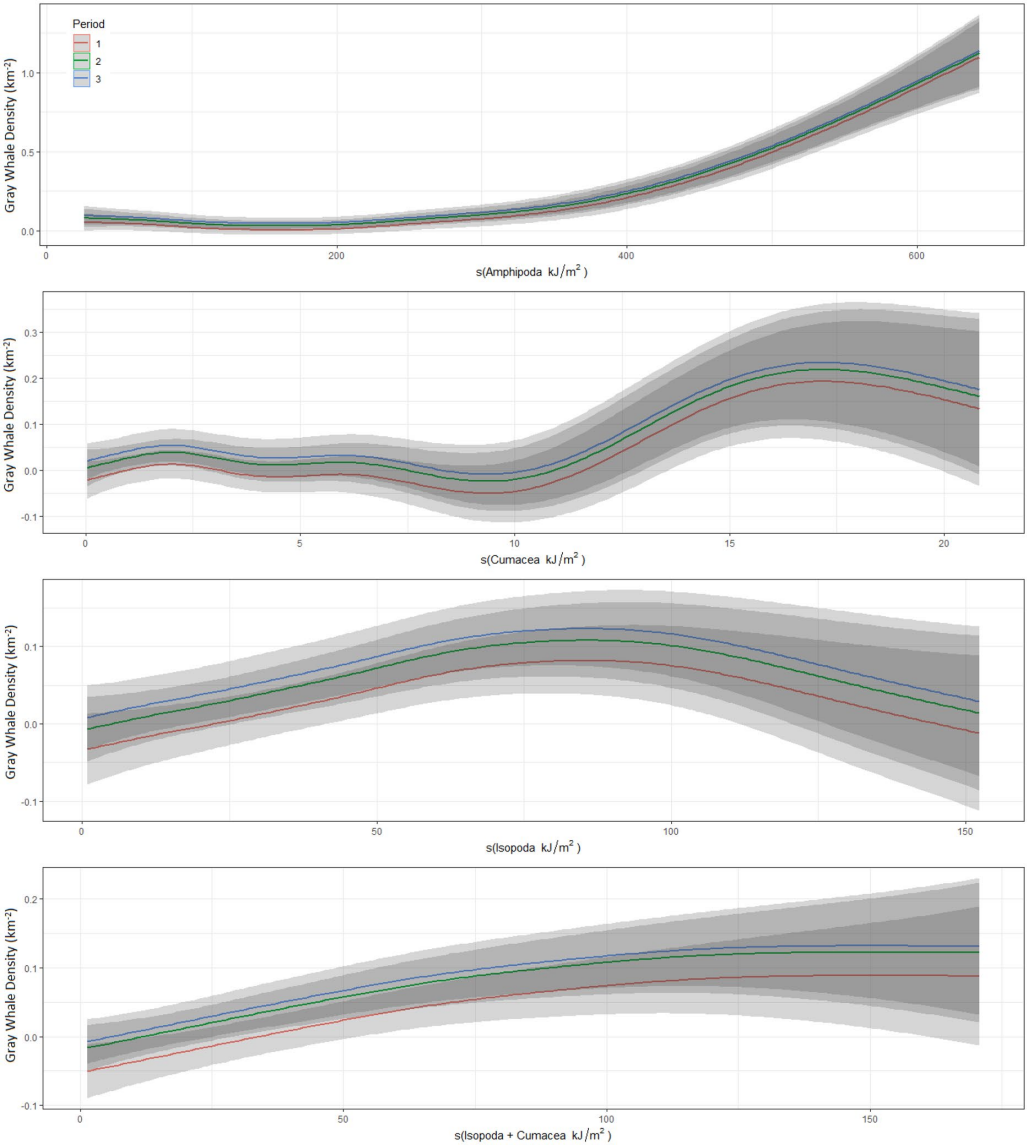
Gray whale density relative to Amphipoda, Cumacea, Isopoda, and a combined Cumacea and Isopoda biomass during different benthic sampling periods in the nearshore feeding area

Gray whale density was significantly lower during the first benthic sampling period (mid-June–mid-July) as whales continued to arrive in the nearshore feeding area. There was no significant difference in mean gray whale density between the second and third sampling period. Gray whale density was significantly higher in areas with higher Amphipoda biomass values, particularly when amphipod biomass values were larger than 300 kJ/m^2^ (Fig. 5). Isopoda and Cumacea energy were also significantly related to gray whale density. Although the relationships between gray whale density and these three species were significant, the patterns were different. For example, the pattern with Amphipoda energy content was more exponential compared to Cumacea or Isopoda. It was also observed that higher energy values of Cumacea were associated with higher energy values for Isopoda. As such, a model containing Amphipoda and the combined Cumacea and Isopoda energy was examined that produced a better fit (lower AIC) compared to the model of each separate species. Among the different benthic periods, the relationship between gray whale density and amphipod energy was significant during periods 2 and 3 compared to period 1. This was likely due to lower number of gray whales in the area or individuals spending more time searching for prey compared to foraging. Both the combined and separate Isopoda and Cumacea relationship with gray whale density were only significant during period 3 compared to the other benthic time periods. The total energy and other prey species (Bivalvia, Actinoptygerii, and Polychaeta) were not significantly associated with gray whale density in 2015. Tables 2–3 provides summary statistics for the whale density and benthic species model.

### Gray whale exposure to vessel sounds

**Fig. 6:**
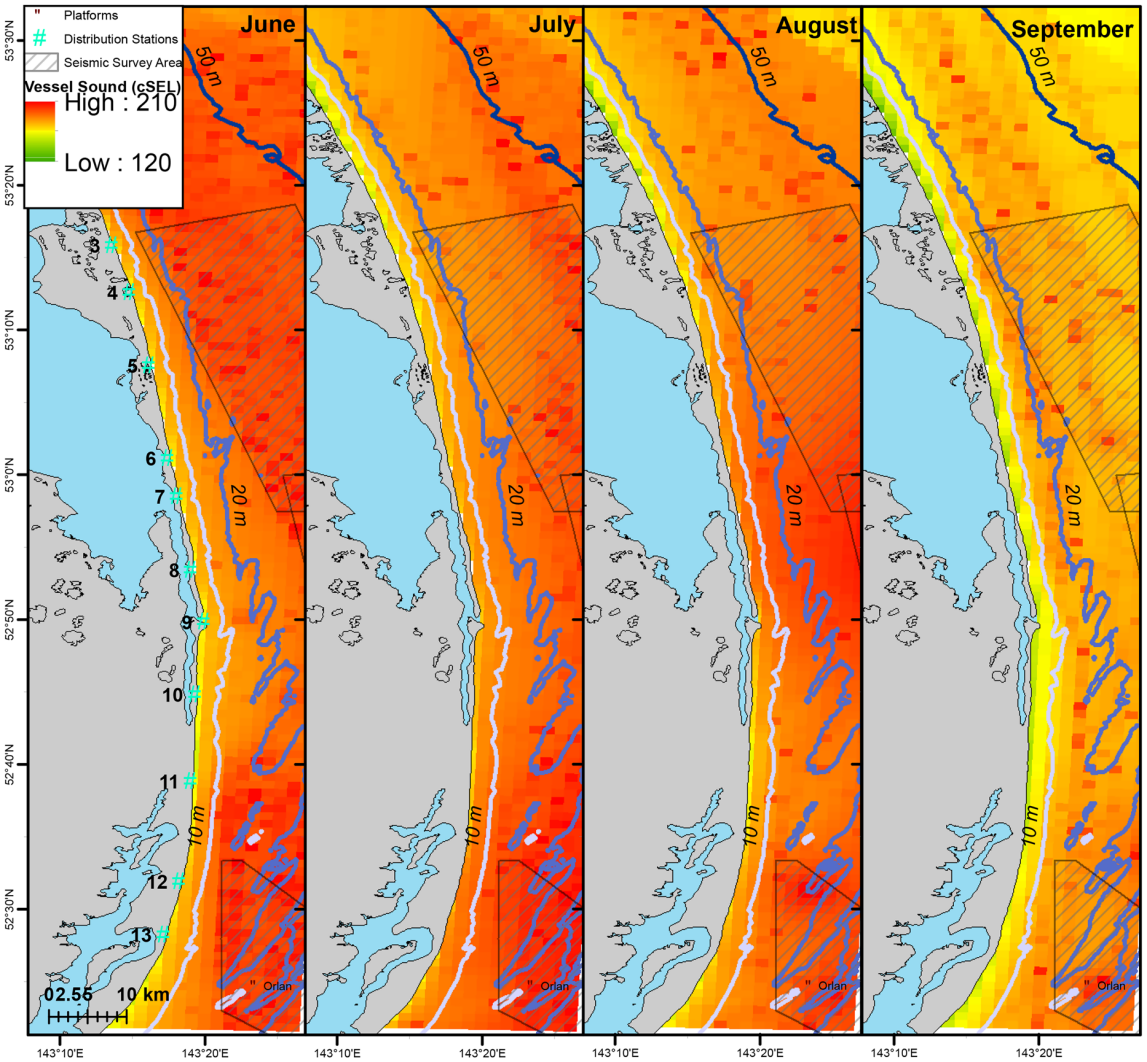
Monthly cumulative sound exposure level (cSEL, dB re 1 μPa^2^s) from vessel activity in the nearshore feeding area of gray whales off Sakhalin Island, Russia, in 2015

**Fig. 7:**
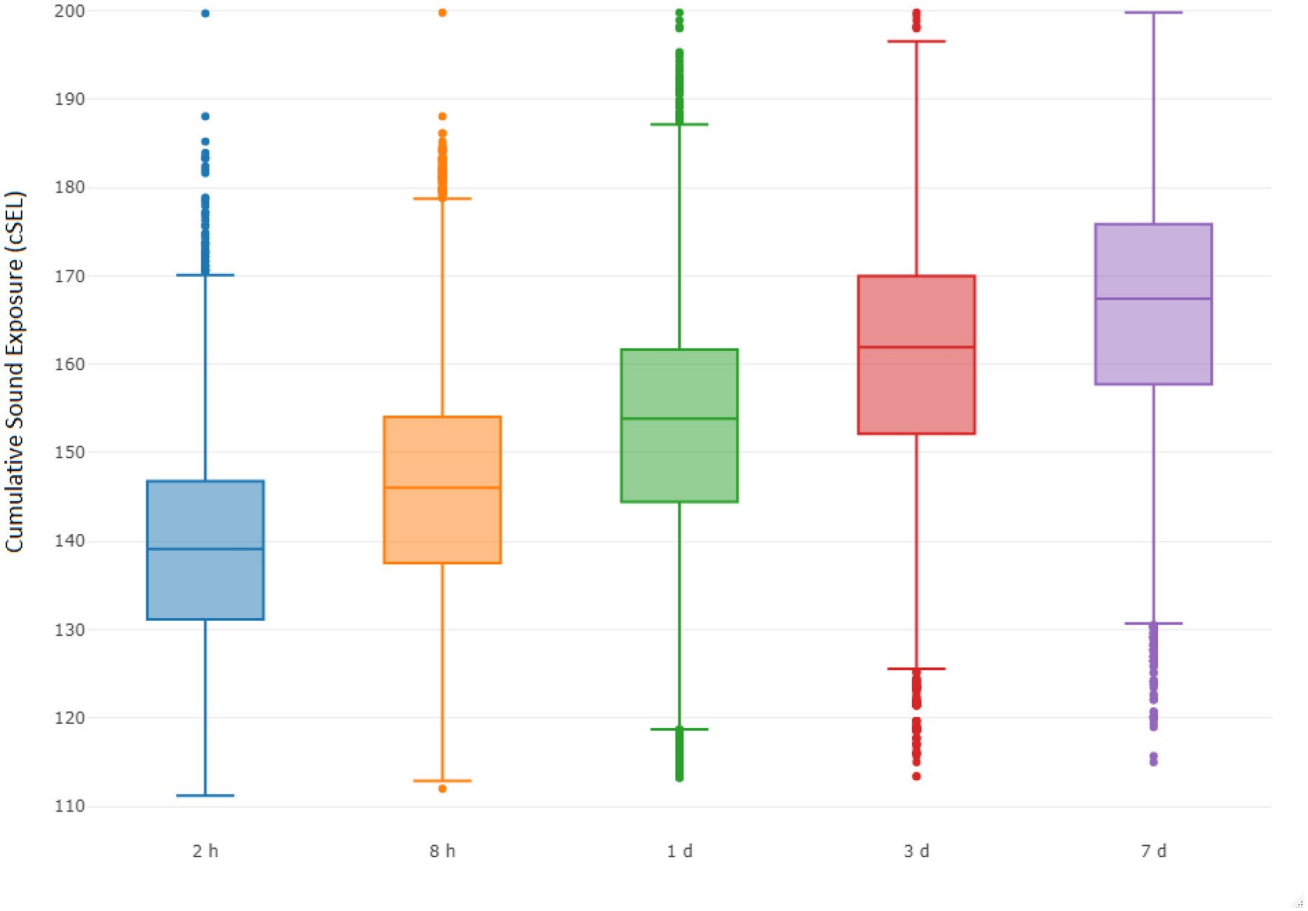
Box-plot of cumulative sound exposure level (dB re 1 μPa^2^s) from vessel activity prior to conducting a distribution scan in grid cells where gray whales were observed

**Fig. 8:**
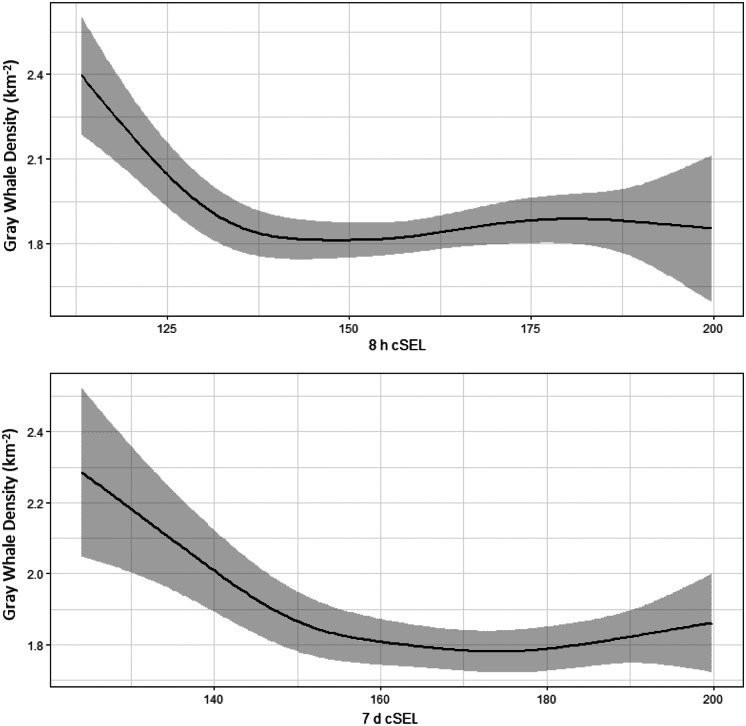
Gray whale density (whales/km^2^) relative to cumulative sound exposure levels (dB re 1 μPa^2^s) from vessel activity in the previous 8 h and 7 d

Vessel activity varied spatially and temporally throughout the gray whale feeding season in 2015. This was partially related to the progression of seismic operations, but also to other activities such as crew change, support, fishing, research vessels, and other vessels that operated throughout the season. The cumulative sound exposure level was highest in the early part of the season and decreased as the field season progressed (Fig. 6). In areas where gray whales were observed, 2 h cSEL values from vessel activity had a mean of 139.1 dB re 1 μPa^2^s (range: 111.2–199.7) and 7 d cSEL values had a mean of 166.5 dB re 1 μPa^2^s (115.0–199.8). Box-plots of the cSEL values for all the computed durations are shown in Fig. 7. Gray whales were found to significantly decrease their density relative to the cumulative sound exposure level in the area over the past 8 h and 7 d (Fig. 8). When the 7 d cSEL was excluded as part of the covariates, the 3 d covariate was the best predictor beyond the initial 8 h response. However, when both 3 d and 7 d were included in the same model, the 7 d covariate accounted for more variation in the response, which suggests that the additional acoustic energy between 3 and 7 d prior to the scan resulted in further decrease in the density of whales in the area. Similarly, the 8 h cSEL accounted for the vast majority of the initial response compared to the 1 d cSEL. These results could suggest that gray whale response to vessel activity could be both short- and longer-term if the activity continued in a particular area over time. On a short-term scale, as a vessel(s) enter(s) an area, some whales could be leaving and coming back shortly thereafter. Over a longer period of time as vessel activity continues, more individuals may leave that area. Table 4 summarizes the best fitting model for whale density and vessel exposure.

### Gray whale exposure to seismic sounds

**Fig. 9:**
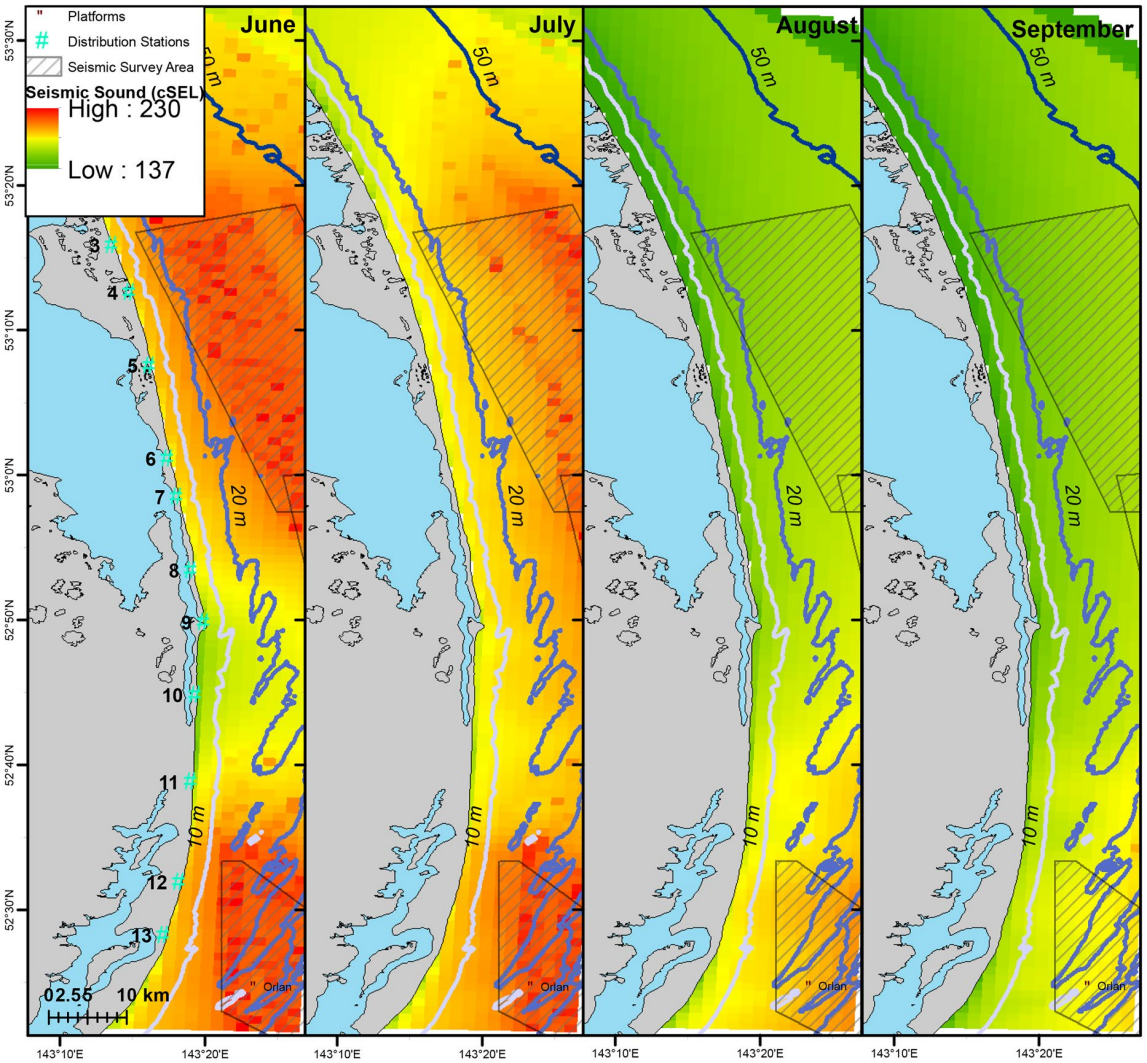
Monthly cumulative sound exposure level (cSEL, dB re 1 μPa^2^s) from seismic activity in the nearshore feeding area of gray whales off Sakhalin

**Fig. 10:**
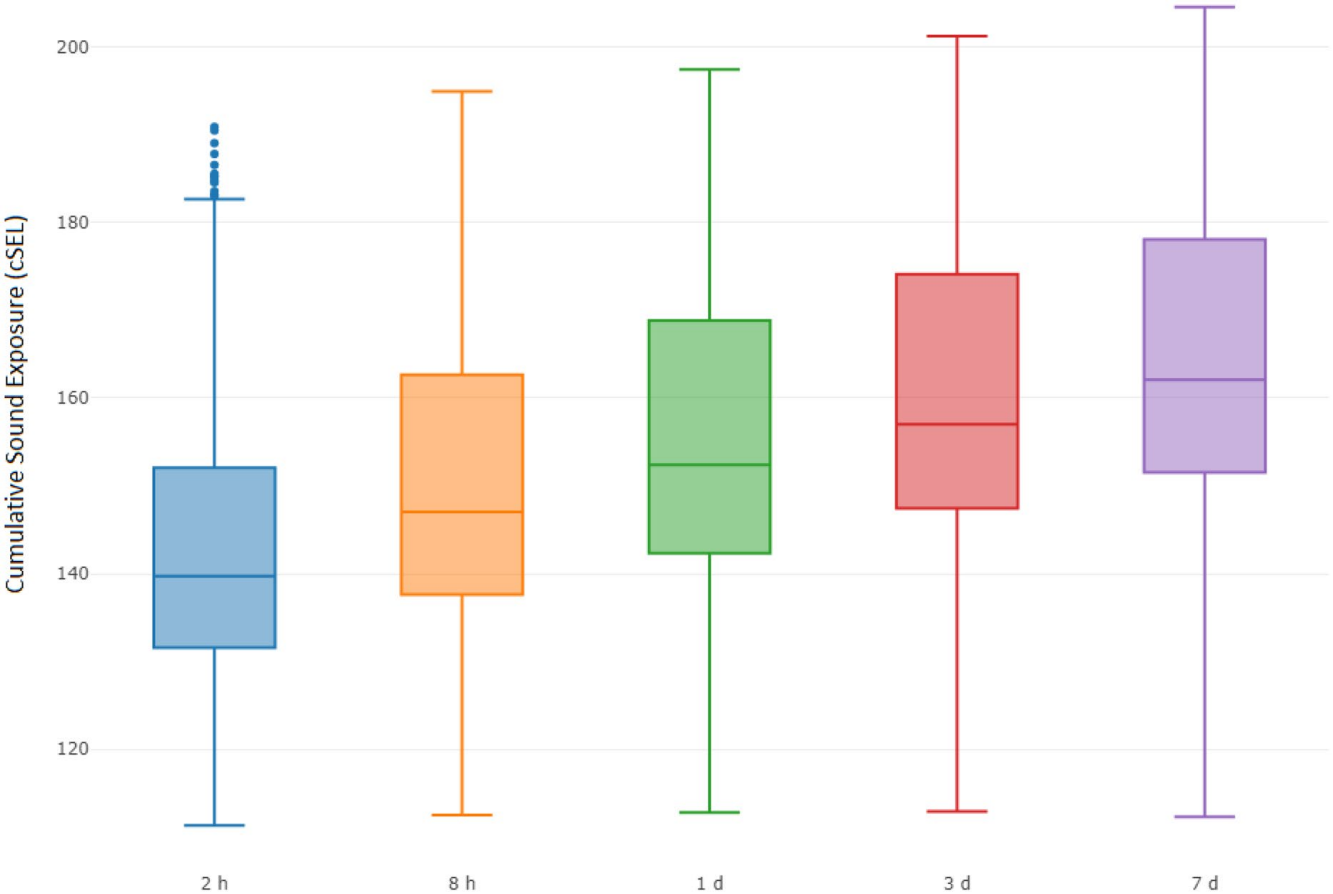
Box-plot of cumulative sound exposure level (dB re 1 μPa^2^s) from seismic sounds prior distribution scans in grid cells where gray whales were observed

**Fig. 11:**
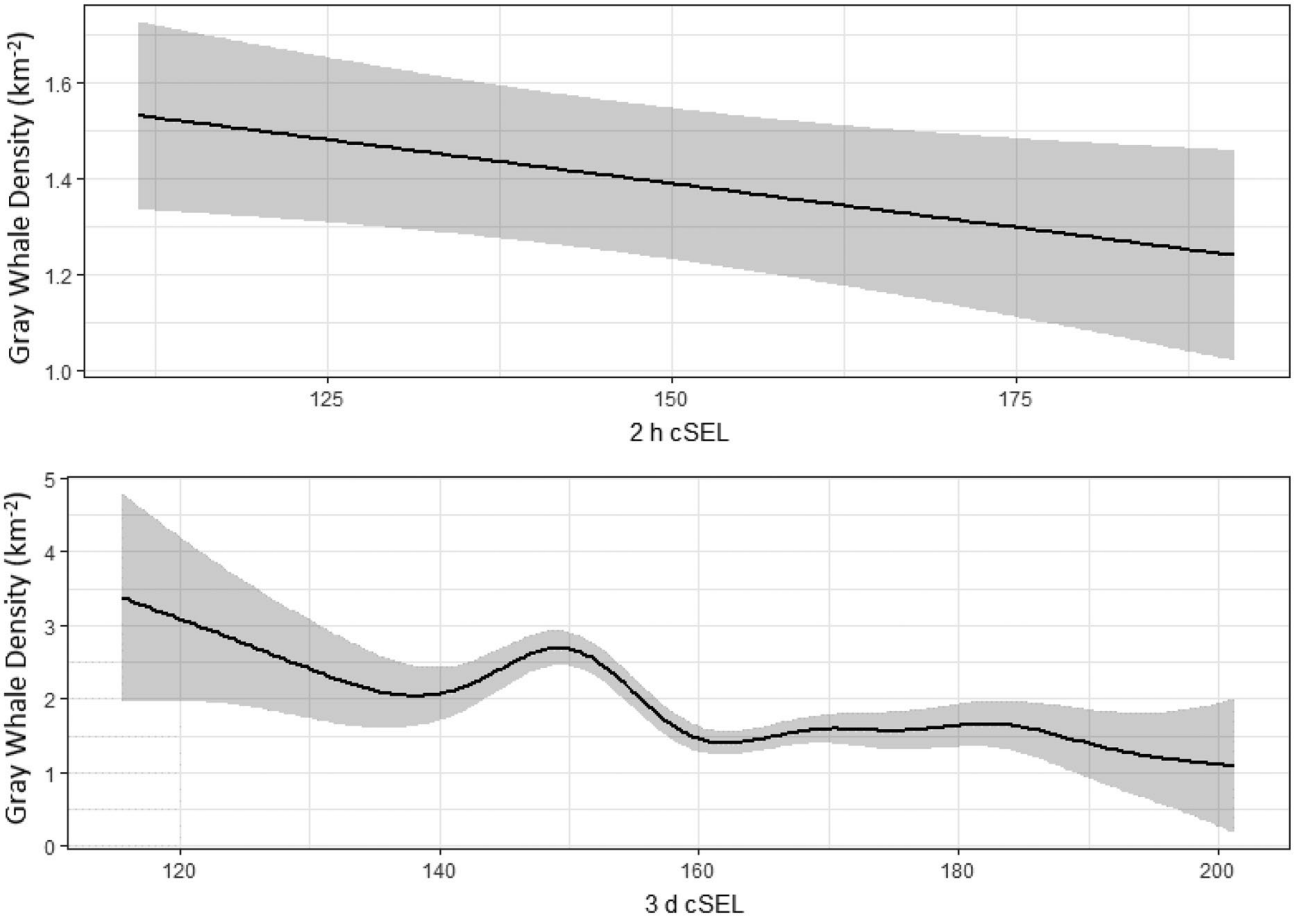
Gray whale density (whales/km^2^) during previous 2 h and 3 d cumulative sound exposure level (dB re 1 μPa^2^s) to seismic sounds

Similar to vessel sounds, seismic exposure varied spatially and temporally due to timing of seismic data acquisition and proximity of the activity to the nearshore feeding habitat. In areas where gray whales were observed, 2 h cSEL values from seismic activity had a mean of 142.2 dB re 1 μPa^2^s (range: 111.3–190.9) and 7 d cSEL values had a mean of 163.8 dB re 1 μPa^2^s (112.3–204.6). Box-plots of the seismic cSEL values for all the computed durations are shown in Fig. 10. As the primary mitigation strategy was to acquire seismic lines closest to the nearshore feeding area as early as possible when fewer gray whales were likely to be present, the greatest amount of exposure occurred in June (Fig. 9). By August, data acquisition in the northern and in most of the central seismic survey areas were completed and considerably less seismic exposure occurred in the nearshore feeding area during the latter part of the feeding season. The best fitting model with gray whale density included the cumulative sound exposure level within the previous 2 h and 3 d (Table 5). On a 2 h time scale, gray whale density decreased slightly when exposed to cumulative sound exposure while an increased decline in whale density occurred on a longer 3 d cumulative exposure (Fig. 10). The longer-term 7 d cumulative sound exposure level was not found to account for additional variation beyond 3 d (Fig. 11). Similar to the vessel activity, gray whales could be responding at shorter and longer time scales to acoustic exposure, but returning to an area after a period of higher exposure occurred.

## Discussion

### Gray whale habitat-use of the nearshore feeding area

Western gray whales migrate over 10000 km to feed within a spatially small foraging ground off Sakhalin Island (Mate et al., 2015). Gray whales observed off Sakhalin have high site fidelity and annual return rate to the two known feeding areas (Bröker et al., 2020). Other foraging areas within the Sea of Okhotsk have not been identified to date. This implies that access to the Sakhalin foraging areas is critical for the survival and reproductive success of the gray whales that feed there. In fact, Gailey et al. (2020) found that poor body conditions and prolonged inter-birth intervals of western gray whales in some years coincided with restricted ability to access the Sakhalin foraging habitat due to sea ice conditions in the previous year. Environmental influences have also been noted to affect calf production and body condition in eastern gray whales (Perryman et al., 2002, 2020; Soledade Lemos et al., 2020).

While on the foraging ground, gray whale distribution and density patterns can be affected by a number of natural factors, such as migration patterns, behavior, prey availability, etc. Early density patterns (May-June) reflect the natural migration of the animals arriving on the foraging ground, with pregnant females generally being the first (~15 May) and mother-calf pairs the last to arrive (~1 July). Gray whale behavior can also influence distribution patterns. For example, individuals arriving in the feeding area early might spend more time searching for high biomass patches, while gray whales arriving later can cue in on the feeding activity of other gray whales. This could partially explain the more uniform distribution observed early in the feeding season in 2015, followed by a more focused and concentrated habitat use pattern. Demographics also influence distribution patterns, with mother-calf pairs and younger individuals being more restricted to the coastline compared to other components of the population (Sychenko, 2011).

In 2015, gray whale densities were observed to increase around mid-June and highest densities occurred around the mouth of Piltun Bay in waters of 10 m or less from early June to the end of September. The nearshore gray whale densities increased rapidly starting from mid-June, reaching a maximum in early August, and started to decline after mid-August. During the course of the feeding season, gray whales were also observed in relative high densities in the northern part of the nearshore feeding area in waters of 20-30 m. These spatial and temporal patterns were captured in the response model with water depth/distance from shore, date, and northing as significant explanatory variables. Although we focused on the utilization of the nearshore feeding area, gray whales also forage offshore Chayvo Bay in the “offshore” feeding area at water depths of 50 m or more. The intra-seasonal changes in density and distribution of the nearshore area can be partially explained by gray whales utilizing one of the foraging areas more than compared to another. Due to the size of the offshore feeding area, vessel expenses, and logistical considerations, sample sizes were drastically smaller compared to the nearshore distribution data, which were collected by relatively inexpensive shore-based techniques. Opportunistic photo-identification studies have, however, demonstrated trends in transitional probabilities between the two feeding areas (Bröker et al., 2020; Schwarz et al., 2022a).

The overall number of gray whales observed to utilize the offshore feeding area has been highly variable over the past two decades. Some individuals, based on photo-identification studies, were shown to preferentially utilize the nearshore feeding area, which included 14% of the known reproductive females that were never photographically captured in the offshore feeding area (Schwarz et al., 2022a). Similarly, mothers with calves have never been observed in the offshore area during the weaning period. Younger individuals (< 5 years of age) also tend to have a higher affinity for the nearshore feeding area (Bröker et al., 2020; Schwarz et al., 2022a). However, the amphipod-rich offshore feeding area (Blanchard et al., 2019; Demchenko et al., 2016) may be critical for pregnant females (McHuron et al., 2021). In 2015, a total of nine vessel-based line-transect surveys were conducted in the offshore feeding area. The number of gray whales observed was higher than those observed in previous years, with almost half the population (~80 individuals) observed in two separate surveys in September. Prior to 2015, higher numbers of western gray whales in the offshore feeding area were also generally observed later in the feeding season. The decreased whale density in the nearshore feeding area later in the season observed during this study was likely partially related to individuals moving to the offshore feeding area and the dynamic interchange of individuals moving between these two feeding areas. Gray whales observed in the offshore feeding area later in the season were likely to have been exposed to higher sound levels compared to the nearshore feeding area due to the proximity to the ongoing seismic activity at the time.

### Gray whale density and their potential prey

Gray whales primarily forage on benthic crustaceans, but they also opportunistically forage on other species that may be high in abundance such as shrimp, crab larvae, mysids, and polychaetes (Brower et al., 2017; Budnikova & Blokhin, 2012; Dunham & Duffus, 2002; Feyrer & Duffus, 2011; Guerrero, 1989; Kim & Oliver, 1989; Moore et al., 2007; Nerini, 1984). Amphipods are considered to be preferential prey due to the high-energy content (Brower et al., 2017; Highsmith & Coyle, 1992; Moore et al., 2003). Gray whale density patterns off Sakhalin in 2015 were significantly associated with the energy content of amphipods, cumaceans, and isopods. This suggests that gray whales primarily foraged on crustacean species and whales did not appear to target patches with high total benthic energy content or energy of other prey species groups (polychaetes, bivalves, and Actinoptygerii (sand lance, *Ammodytes hexapterus*)). The gray whale density model that included a combined energy of isopods and cumaceans included with amphipods explained more variation in whale density than all other models considered. Gray whales may have been taking advantage of the higher energy content of both species groups instead of targeting one particular group, but gray whale densities were generally higher in areas that contained amphipod energy content greater than 300 kJ/m^2^. Stomach content studies of gray whales reflect the faunal composition in their feeding area (Budnikova & Blokhin, 2012; Nerini, 1984) with bivalves and other animals likely to be incidental catches. Actinoptygerii, particularly sand lance, have also been suggested to influence western gray whale habitat use within their nearshore feeding grounds (Blanchard et al., 2019). It is possible that western gray whales observed in the northern part of the feeding area in >20 m depths foraged on sand lance, but the benthic sampling period (a month or longer) could not capture the localized, shorter-term foraging patterns with this group. Actinoptygerii would only become available to whales when the species migrates to and aggregates in the feeding area, and this periodic migration was not adequately captured in our benthic study (Blanchard et al., 2022a).

### Influence of seismic and vessel sounds on gray whale density

The influence of vessel and seismic sounds on gray whale behavior, density, and distribution has been noted in previous studies off Sakhalin. During a 2001 seismic survey, gray whale abundance significantly decreased in the area with highest sound exposures and multiple indicators of behavioral response to the activity were observed (Gailey et al., 2007; Weller et al., 2002; Yazvenko et al., 2007). In contrast, analyses from a seismic survey in 2010 found only slight indications of gray whale density, distribution, and behavioral responses (Gailey et al., 2016; Muir et al., 2015a, 2016), likely due to the small sample size that limited detection of responses.

In 2015, the number and duration of seismic operations exceeded those from previous years. Due to the monitoring design and duration of seismic activity (Aerts et al., 2022), sample sizes drastically increased allowing detection of subtle-to-moderate changes in gray whale density relative to seismic and vessel activity. Both vessel and seismic cumulative sound exposure levels in periods prior to the distribution scan were found to be associated with decreased whale densities after accounting for spatial and temporal patterns in gray whale habitat use. For both vessel and seismic sounds, higher cumulative sound exposure level decreased whale density during shorter (2 h seismic, 8 h vessel) exposure periods. After accounting for the density response for the shorter exposure periods, additional exposure levels further decreased gray whale density at longer exposure periods of 3 d (seismic) and 7 d (vessel). This suggests gray whales responded more variably to short-term exposure while increased anthropogenic activity in a particular area over longer periods decreased the density of whales in the area. Individual heterogeneity is likely to play an important role in gray whale responses as some individuals (potentially of a particular demographic group) could be more sensitive to exposure while other individuals may tolerate shorter-term exposure more.

Seismic surveys not only expose animals to pulsed sound levels, but also increase the amount of vessel activity in the area due to the multiple vessels required during a seismic operation. In addition, other vessels (e.g., support, crew change, research, fishing, shipping) operate in the area and contribute to the overall soundscape. Eastern gray whales have also been shown to respond to vessel activity in their breeding lagoons (Bryant et al., 1984). Gray whales are also likely exposed to heavy vessel traffic throughout their home range with the potential risk to their survival associated with ship strikes (Silber et al., 2021). Western gray whales have been observed to respond behaviorally to the proximity and sound levels of vessel activity in the study area (Gailey, 2013; Gailey et al., 2007, 2016, 2022). Although gray whales were found to respond to both seismic and vessel activities, some gray whales remained in the nearshore feeding area even during periods of relatively high sound exposure. Leaving a foraging area that sustains survival might be energetically costly and individuals could therefore be resistant to respond. Some individuals may be more habituated to seismic and vessel activity while other individuals could be sensitized to the activity due to previous negative or novice experience to sound exposure. Alternatively, individual gray whales could tolerate the activity and remain in the area due to their need to forage or to avoid predation pressure. Other physiological responses, such as stress, could also be impacting the whale over the long-term (Bejder et al., 2009; Blumstein, 2016). Gailey et al. (2022) found significant respiration responses relative to the activities that could indicate animals are physiologically responding as well. Prolonged and repeated stress responses can have serious conservation implications (Wright et al., 2007, 2011), but limited information is available on how marine mammals could be affected. With a population that has such high site fidelity to a small geographic foraging area, there may be nowhere to go during intense periods of anthropogenic activity, particularly for mother-calf pairs and younger individuals in this population (Forney et al., 2017).

The vast majority of the closest (highest exposure) seismic activity occurred early in the feeding season (June), while by August most of the activity was restricted to the southern part of the nearshore feeding grounds where gray whale abundance and prey biomass tended to be lower. Comparative to limited access to early foraging due to sea ice conditions, restricted access early in the foraging season could have the greatest impacts on pregnant females who have the highest energetic demand during the foraging season. Several studies have suggested that restricted access to the foraging area due to environmental factors likely impacts calf production and reproduction rates (Gailey et al., 2020; Perryman et al., 2002, 2020; Salvadeo et al., 2015). However, McHuron et al. (2021) found that limited access to the feeding area early in the season would have no effect on pregnant females’ reproductive success.

Gray whale responses to anthropogenic activities in 2015 as documented in this study and in Gailey et al. (2022) could potentially result in biologically significant impacts in the longer term, such as lower reproductive rates or survival. In 2015, a minimum of 14 pregnant females were present on the Sakhalin foraging ground, based on the number of observed calves in 2016. As of 2020, only three of those calves (21%) have been resighted and thus known to have survived. With 210 potential foraging days in 2015, based on sea ice conditions within their feeding ground (Gailey et al., 2020), the apparent calf survival of pregnant females in 2015 was significantly lower than those observed with similar foraging durations in previous years (Gailey et al., 2020). Loss of potential foraging days due to repeatedly responding to disruptive activities could have prevented pregnant females from gaining enough fat reserves to sustain them and wean their calves. Declining prey resources in the nearshore feeding area (Blanchard et al., 2019) may also have contributed to the lower apparent calf survival, as those calves could have been weaned at a lower mass. Of the calves observed in 2015 (11 calves, 208 feeding days in 2014), five (46%) have been observed to have apparently survived as of 2020. Calf survival in 2015 was within the normal range of apparent calf survival off Sakhalin (Gailey et al., 2020), which implies that pregnant females in 2014 were able to obtain enough fat reserves.

The number of known seismic surveys near the western gray whale feeding areas has increased in recent years from one seismic survey during 2000–2009 to at least five feeding seasons with seismic surveys from 2010-2019. This increasing seismic survey activity level elevated concerns about disturbance to gray whale foraging activities and the potential for biologically significant impacts on the population. Although behavioral and distributional responses to seismic and vessel sounds have continually been observed, it remains unknown to what extent these responses will have biologically significant effects on individuals or the population, especially in the long term. Western gray whales have numerous stressors impacting their annual survival, both of natural (e.g., predation, food availability, environmental) and anthropogenic origin (e.g., oil and gas activities, fishery, tourism, vessel traffic). The effects of the anthropogenic activities should not be viewed in isolation but as a cumulative impact relative to other known and unknown factors impacting the future survival of the western gray whale population.

This study examined gray whale habitat use of the nearshore feeding area while individuals were exposed to seismic and vessel sounds in a single feeding season. Components of the population, such as reproductive females or young, could be under-represented in the analyses. For example, if reproductive females were particularly sensitive to anthropogenic activity, their responses would be harder to detect as they represent a small portion (19%) of the population. Density responses noted here could be indicative of a larger response by a particular demographic component of the population. Future studies should focus on heterogeneity of the responses among different components of the population both spatially and temporally. A better understating of the contextually explicit responses among individuals would likely improve our understanding of the effects of disturbance to individuals, demographic groups, and the population (Pirotta et al., 2018, 2021).

## Appendix

**Table 1:** Summary of the best fitting GAM model between gray whale density and benthic energy that included a combined energy between Isopoda and Cumacea

**Variable**	**edf**	**Ref.df**	**F**	**p-value**
s(Depth)	5.355	9	9.79	0.0000
s(Northing)	3.724	9	3.27	0.0000
s(Amphipoda):Period2	4.777	9	14.75	0.0000
s(Amphipoda):Period3	6.142	9	8.12	0.0000
s(Isopoda+Cumacea):Period2	0.498	9	0.11	0.1487
s(Isopoda+Cumacea):Period3	7.558	9	11.99	0.0000

**Table 2:** Summary of the best fitting GAM model between gray whale density and benthic energy

**Variable**	**Estimate**	**Std. Error**	**t value**	**Pr(>|t|)**
(Intercept)	0.064	0.006	10.76	0.0000
Period 2	0.011	0.011	0.94	0.3484
Period 3	0.004	0.009	0.45	0.6553

	**edf**	**Ref. df**	**F**	**p-value**
s(Depth)	5.160	9	9.25	0.000
s(Northing)	3.711	9	3.04	0.000
s(Amphipoda):Period2	4.821	9	14.89	0.000
s(Amphipoda):Period3	6.049	9	4.43	0.000
s(Cumacea):Period2	0.000	9	0.00	0.3134
s(Cumacea):Period3	3.905	9	1.48	0.0043
s(Isopoda):Period2	0.738	9	0.19	0.0989
s(Isopoda):Period3	8.480	9	9.30	0.000

**Table 3:** Summary of the best fitting GAM model between gray whale density and natural explanatory variables

**Variable**	**edf**	**Ref.df**	**F**	**p-value**
s(DistShore)	0.064	9	820.90	0.0000
s(Depth)	0.011	9	829.89	0.0000
s(Northing)	0.004	9	952.69	0.0000
s(Date)	7.457	9	49.34	0.0000

**Table 4:** Summary of the best fitting GAM model between gray whale density and vessel sounds

**Variable**	**edf**	**Ref.df**	**F**	**p-value**
s(DistShore)	7.973	9	636.96	0.0000
s(Northing)	8.734	9	873.45	0.0000
s(Date)	7.684	9	38.84	0.0000
s(Vessel_cSEL_8h)	6.573	9	15.79	0.0000
s(Vessel_cSEL_168h)	8.882	9	157.44	0.0000

**Table 5:** Summary of the best fitting GAM model between gray whale density and vessel sounds

**Variable**	**edf**	**Ref.df**	**F**	**p-value**
s(DistShore)	7.977	9	845.86	0.0000
s(utmy)	8.915	9	651.79	0.0000
s(date)	7.241	9	25.95	0.0000
s(Med_cSEL_2h)	2.438	9	2.18	0.0000
s(Med_cSEL_72h)	7.941	9	151.15	0.0000
